# P02-10 Irish primary school teacher's perceptions and practices of physical education provision

**DOI:** 10.1093/eurpub/ckac095.029

**Published:** 2022-08-29

**Authors:** Mairéad Teehan, Lisa Kelly, Aoife Lane, Niamh Ní Cheilleachair, Kieran Dowd

**Affiliations:** Department of Sport and Health Sciences, TUS Midlands, Athlone, Ireland

**Keywords:** primary school, physical education

## Abstract

**Background:**

Primary school physical education (PE) is critical for the development of skills that are essential for lifelong participation in physical activity (PA). Despite this, PE provision in Irish schools is inconsistent, while just 17% of Irish children currently achieve the recommended 60 minutes of daily PA. PE exposes children to regular, developmentally appropriate PA and teachers are essential in ensuring high quality PE is provided. This study aims to examine the perceptions and practises related to PE amongst Irish primary school teachers.

**Methods:**

A survey was developed and validated using a modified Delphi technique and included 57 questions to examine Irish primary school teachers current teaching practices and supports needed to enhance their PE provision. A link to the survey was emailed to all Irish primary schools and shared on social media. SPSS version 27 was used for data analysis.

**Results:**

Of the 473 respondents, 84.4% were female, 15.2% male and 0.4% didn't say (median age=34 (IQR=12)). Respondents indicated PE is timetabled (90.7%) and taught weekly (97%) in most schools. Time allocated for PE is used for other subjects in 35.9% of cases. Respectively, 40.8% and 52.4% of respondents indicated they never plan for or reflect on their teaching of PE. Just 57.1% of respondents indicated sufficient facilities, while 46.3% believe their equipment is insufficient. Approximately half (45.5%) have undertaken continuous professional development, citing a lack of courses offered, time and other priorities as barriers to undertaking additional training. Teachers cited their own lack of competence and confidence to teach PE as reasons why they would welcome engagement from a specialist PE teacher for certain strands (32.3%) or as a general support in PE (24.1%).

**Conclusion:**

Although the vast majority of primary school teachers deliver PE on a weekly basis, these findings suggest teachers require additional supports to assist in their provision of PE. Novel approaches, such as the engagement of specialist PE teachers to support delivery in primary schools, are required to improve PE for Irish youths.

